# The Concentration of PCSK9-Lp(a) Complexes and the Level of Blood Monocytes in Males with Coronary Atherosclerosis

**DOI:** 10.3390/jpm13071077

**Published:** 2023-06-29

**Authors:** Anastasiia Yu. Filatova, Olga I. Afanasieva, Tatiana I. Arefieva, Alexandra V. Potekhina, Alexandra V. Tyurina, Elena A. Klesareva, Oksana A. Razova, Marat V. Ezhov, Sergey N. Pokrovsky

**Affiliations:** 1 Institute of Experimental Cardiology, National Medical Research Center of Cardiology Named after Academician E.I. Chazov, Ministry of Health of the Russian Federation, 121552 Moscow, Russia; afanasieva.cardio@yandex.ru (O.I.A.); tiarefieva@cardio.ru (T.I.A.); hea@mail.ru (E.A.K.); razova1@yandex.ru (O.A.R.); dr.pokrovsky@mail.ru (S.N.P.); 2 A.L. Myasnikov Institute of Clinical Cardiology, National Medical Research Center of Cardiology Named after Academician E.I. Chazov, Ministry of Health of the Russian Federation, 121552 Moscow, Russia; potehina@gmail.com (A.V.P.); alex.tyurina.cardio@yandex.ru (A.V.T.); marat_ezhov@mail.ru (M.V.E.)

**Keywords:** lipoprotein(a), PCSK9, PCSK9-Lp(a), monocytes, atherosclerosis

## Abstract

In this study we analyzed the concentration of lipoprotein(a) (Lp(a)), PCSK9-Lp(a) complexes and the circulating monocyte subsets in coronary atherosclerosis. For this study, 257 patients with coronary atherosclerosis and 68 patients without stenotic atherosclerosis in the coronary, carotid and lower extremity arteries (control group) were enrolled. The monocyte subpopulations (classical CD14++CD16-, intermediate CD14++CD16+ and non-classical CD14+CD16++) were analyzed by direct immunofluorescence and flow cytometry. The Lp(a) and PCSK9-Lp(a) complexes in the serum were detected by ELISA. The concentration of Lp(a) was higher in the coronary atherosclerosis group compared with the controls (23.0 (9.1; 73.3) mg/dL versus 10.7 (4.7; 25.0) mg/dL, *p* < 0.05). No correlations between the level of Lp(a) and the concentration of the PCSK9-Lp(a) complexes, nor between the level of Lp(a) or PCSK9 and the total number of monocytes, were observed in either group. A slight positive correlation between the concentration of PCSK9-Lp(a) complexes and the absolute level of monocytes was obtained (r = 0.20, *p* = 0.002) in the patients with atherosclerosis due to the intermediate monocyte subsets (r = 0.33, *p* = 0.04). According to regression analysis, both the PCSK9-Lp(a) complexes concentration and BMI were related to the absolute number of blood monocytes in patients with atherosclerosis. Further studies are required to determine the pathogenetic contribution of PCSK9-Lp(a) complexes to the development of atherosclerosis.

## 1. Introduction

Atherosclerosis is currently considered a chronic inflammatory process in the intima of the arterial wall due to endothelial dysfunction accompanied by the accumulation of modified lipoproteins, products of cell and tissue decay as well as other substances. Lipoprotein(a) (Lp(a)) is a supramolecular complex consisting of a low-density lipoprotein (LDL)-like apoB-100-containing particle and a highly glycosylated apolipoprotein(a) molecule [1]. An elevated blood concentration of Lp(a) is a defined genetic risk factor for atherosclerotic cardiovascular disease and its complications. Currently, there are many published data showing the association of a high concentration of Lp(a) with early manifestation and the accelerated development of atherosclerosis of various vascular basins as well as the higher incidence of adverse cardiovascular events [2,3,4]. Patients with coronary artery disease and elevated Lp(a) levels taking statins who underwent percutaneous coronary intervention showed a higher frequency of major adverse cardiovascular events in a long-term period [5,6]. Moreover, the meta-analysis of Willeit P. et al. [7] demonstrated a stronger association of an increased level of Lp(a) with the development of cardiovascular events in patients receiving statins after the adjustment of traditional risk factors, compared with patients not taking statins. These data confirm the contribution of Lp(a) to the development of cardiovascular complications as a residual factor.

The mechanisms underlying the high atherogenicity of Lp(a) are poorly understood [8]. Lp(a) has additional pro-inflammatory and atherothombogenic properties due to the presence of apolipoprotein(a) (apo(a)) and its structural homology with plasminogen molecules [1]. According to the earlier described heterogeneity in apo(a) size associated with the copy number variation in the kringle IV type 2 protein domain, apo(a) isoforms are conveniently classified into low molecular and high molecular weight phenotypes. It seems that the low molecular weight apo(a) phenotype is more atherogenic than the high molecular weight phenotype. Analysis of 40 studies showed that patients with the low molecular weight apo(a) phenotype had a relative risk two times higher for coronary heart disease compared with individuals with high molecular weight isoforms [9]. It has been shown that Lp(a), through oxidized phospholipids bound to its apolipoprotein(a), contributes to monocyte activation and endothelial dysfunction [10]. According to Schnitzler J. [11], Lp(a)-stimulated endothelial cells mediated by 6-phophofructo-2-kinase/fructose-2,6-biphosphatase-3 glycolysis and activated inflammatory pathways increase monocyte adhesion and migration. In experiments with mice, incubation of C57BI6 bone marrow cells with Lp(a) resulted in increased production of granulocyte-monocyte progenitors and differentiation into pro-inflammatory Ly6^high^ monocytes [12], while antibody-mediated neutralization of oxidized phospholipids reversed these effects. In addition, Lp(a) is able to bind pro-inflammatory molecules and shuttle them to the atherosclerotic plaque, thereby maintaining inflammation in the vessel wall [13,14].

Monocytes and macrophages are the major immune cell populations involved in atherogenesis. Monocytes migrate to the sub-endothelial space due to signals received from damaged tissues and further differentiate into macrophages. In the early stages of the development of atherosclerotic plaque, monocytes enter the arterial intima through the luminal endothelial monolayer, but when the plaque matures, monocytes may enter the plaque through neovascularization. In the vessel wall, macrophages transform into foam cells. Foam cells aggregate to form the atheromatous core, and subsequently the atheromatous centers of the plaque become necrotic, consisting of lipids, cholesterol crystals and cell debris [15]. Accumulated in macrophages, cholesterol crystals can induce NLRP3 inflammasome activation, resulting in secretion of interleukin-1 family cytokines. Thereby, macrophage-secreted cytokines and other mediators (active oxygen species, proteolytic enzymes, etc.) fuel the chronic vascular inflammation that drives atherosclerosis [16].

Depending on the expression of CD14 and CD16 surface markers, blood monocytes are divided into CD14++CD16- classical, CD14++CD16+ intermediate and CD14+CD16++ non-classical subsets [17]. In patients with atherosclerosis and other chronic inflammatory diseases, the amount and composition of the circulating monocytes is altered [18]. In particular, the content of CD16+ intermediate monocytes, demonstrating the most pronounced pro-inflammatory activity, is increased [19,20]. According to our previous data, the level of circulating intermediate monocytes is associated with the severity of atherosclerosis in younger (<60 years old) patients but not in older patients with atherosclerotic cardiovascular disease [21].

The hypothesis that high atherothrombogenicity of Lp(a) is associated with the “inflammatory” component and the involvement of the immune system, particularly blood monocytes, is currently relevant [22]. Krychtiuk K.A. et al. showed an increased relative content of circulating intermediate monocytes in patients with stable coronary artery disease and elevated Lp(a) levels [23]. We have also shown an association between Lp(a) levels and an elevated amount of circulating CD16+ monocytes (both intermediate and non-classical) in patients with stenotic three-vessel coronary artery disease [24].

Proprotein convertase subtilisin/kexin type 9 (PCSK9), which is involved in the degradation of LDL receptors on hepatocytes, regulates the blood levels of LDL cholesterol [25]. Recent studies confirmed the association of PCSK9 levels with the occurrence of cardiovascular events [26], regardless of adequate pharmacological control of LDL cholesterol and correction of the traditional risk factors for atherosclerosis [27]. The use of PCSK9 inhibitors significantly reduces the risk of cardiovascular disease [28,29], with a robust effect in patients with elevated Lp(a) regardless of the LDL-C achieved [28]. These data suggest additional Lp(a)-mediated mechanisms for the pro-atherogenic (pro-inflammatory) effects of PCSK9.

Along with hepatocytes, PCSK9 is also synthesized by endothelial cells, smooth muscle cells and macrophages [30,31,32]. Emerging evidence suggests an immunomodulatory effect from PCSK9 by stimulating the differentiation of lymphocytes with pro-inflammatory properties. An experimental study by Kim et al. [33] showed higher blood levels of interleukin-17 and a predominance of T-cell differentiation toward T helper 17 in atherosclerotic hypolipidemic Ldlr^−/−^Apobec1^−/−^ (LDb) mice. Additional PCSK9 gene knockout in these mice was associated with significantly lower plasma interleukin-17 levels as well as T helpers 17 and CD4+ memory T cell levels in the spleen and RORC mRNA expression compared with LDb mice. Incubation of T cells isolated from carotid plaque samples after endarterectomy or from the peripheral blood of healthy donors with oxidized LDL-treated dendritic cells (for PCSK9 induction) was accompanied by lymphocyte proliferation, with preferential differentiation into T helper 17 and T helper 1, and the production of interferon-γ and interleukin-17. PCSK9 inhibition prevented the effects of oxidized LDL on the dendritic cells and T-lymphocytes [34]. According to several studies [35,36], PCSK9 binds to the CD36 scavenger receptor and regulates its expression in macrophages, enabling this molecule to be considered for a potential damage-associated molecular pattern [37].

Lp(a) has been shown to form complexes with PCSK9 [38]. Furthermore, PCSK9 in complex with Lp(a) [38] is the dominant pool of lipoprotein-associated PCSK9. We hypothesized that Lp(a) may exert its proatherogenic activity via PCSK9. In this study, we analyze the concentrations of Lp(a) and PCSK9-Lp(a) complexes and the circulating monocyte subsets in males with atherosclerotic cardiovascular diseases and in patients without atherosclerosis.

## 2. Materials and Methods

This study was approved by the Institutional Ethics Committee and carried out in accordance with the principles of the Declaration of Helsinki. Written consent was obtained from each patient. In total, 257 males with coronary atherosclerosis verified by coronary angiography were screened. The exclusion criteria were the following: acute coronary syndrome or interventions in the previous 6 months, a history of stroke, neoplasms, liver or renal failure, infectious or inflammatory diseases or decompensated diabetes mellitus, current use of immunosuppressive drugs and statin therapy for less than 6 months prior to enrolment in the trial. Arterial hypertension was diagnosed if the patient received antihypertensive treatment or in cases of the systolic blood pressure level being above 140 mmHg or diastolic blood pressure being above 90 mmHg according to two blood pressure measurements on two different visits. The smoking status was determined as never a smoker or a former or current smoker. Type 2 diabetes was diagnosed according to the World Health Organization criteria [39]. The body mass index (BMI) was calculated for all the participants, and obesity was recorded at BMI ≥ 30 kg/m^2^.

A total of 68 consecutive male patents who underwent clinical examination (from 2020 to 2022) and demonstrated the absence of stenotic atherosclerosis in the coronary, carotid and lower extremity arteries, as confirmed by coronary angiography and ultrasound duplex scanning of the carotid and femoral arteries, comprised the control group. Patients in the control group were not on statins.

Coronary angiography was performed via a transradial approach using a standard technique. The presence of coronary atherosclerosis was assessed with the degree of stenosis of the main coronary arteries. The severity of coronary atherosclerosis was assessed in the projection with the greatest degree of stenosis by one experienced independent observer. Stenotic atherosclerosis was identified as narrowing of the artery lumen by more than 50%.

All patients underwent ultrasound duplex scanning of the carotid and femoral arteries. Duplex scanning of the carotid arteries was performed using a high-resolution ultrasound system with a linear array transducer (3–9 MHz). The degree of stenosis of the carotid and femoral arteries was assessed as the total percentage of stenosis.

The concentrations of total cholesterol (TC), triglycerides (TG) and high-density lipoprotein cholesterol (HDL-C) were measured using the enzymatic colorimetric method on Hitachi 912 biochemical analyzers (Roche Diagnostics, Basal, Switzerland) and an Architect C-8000 analyzer (Abbott, Abbott Park, IL, USA). The quality control of the studies was accomplished with the control sera Precinorm and Precipat (Roche Diagnostics, Basal, Switzerland). The low-density lipoprotein cholesterol (LDL-C) was calculated using the Friedewald formula with modifications [40]: LDL-Ccorr (result in mmol/L) = TC − HDL-C − TG/2.2 − 0.3 × Lp(a) mass (mg/dL)/38.7, where LDL-Ccorr is the level of LDL-C corrected to the level of Lp(a) cholesterol. The Lp(a) concentration was measured with an enzyme-linked immunosorbent assay (ELISA) using monospecific polyclonal sheep antibodies against human Lp(a) as previously described [41]. The sensitivity of the method was 0.2 mg/dL, the intra-plate and inter-experiment variation coefficients were 3.8% and 9.8%, respectively, in the Lp(a) concentration ranging from 5 to 190 mg/dL. The method was validated with two kits: TintElize Lp(a) (Biopool AB, Umea, Sweden) and Immunozym Lp(a) (Progen Biotechnik GmbH, Heidelberg, Germany). The control serum (Technoclone, Vienna, Austria) was approved by the International Federation of Clinical Chemistry and was used to standardize the ELISA. The results were obtained from a Multiscan Go microplate spectrophotometer (Thermo Scientific, Vantaa, Finland). The concentration of PSCK9 was measured by an ELISA using a Quantikine ELISA Human Proprotein Convertase 9/PCSK9 Immunoassay commercial kit (R&D Systems, Minneapolis, MN, USA). The concentration of PSCK9-Lp(a) complexes was detected by an ELISA with laboratory monoclonal antibodies against PCSK9 and polyclonal antibodies against Lp(a) [42]. A preparation of human monoclonal antibodies against PCSK9 (140 mg/mL; Amgen) was diluted with 10 mM of a phosphate buffer (pH of 7.4) to a final concentration of 100 µg/mL. The ELISA plates (Costar) were incubated for 1 h at 37 °C and then for 16 h at 4 °C with 100 μL of solution in each well. Further analysis was carried out similarly to the measurement of the Lp(a) concentration. The test samples were first diluted by 10 times, and then serial 30-fold dilutions were prepared. To analyze the cross-reactions, samples of PCSK9 (standard protein solution with a protein concentration of 40 ng/mL; R&D Systems) were used [42].

Blood samples were obtained from all patients before coronary angiography. Whole blood was collected in a sodium citrate antiocoagulated vacutainer tube. The samples were processed within 2 h after being collected. Monocyte immunophenotyping was performed in the blood samples by direct immunofluorescence using fluorescently labeled antibodies against CD45, CD14 and CD16 antigens (Beckman Coulter, Brea, CA, USA) and a FACS lysing solution (BD Immunocytometry Systems) according to the manufacturer’s instructions. The samples were analyzed on FACS Calibur and FACS Canto flow cytometers (BD Immunocytometry Systems). Monocytes were gated according to the light scatter parameters and CD45 expression pattern. Monocyte subsets were identified as classical (CD14++CD16−), intermediate (CD14++CD16+) and non-classical (CD14+CD16++) according to the routinely used protocol [17].

Statistical analysis was performed using the MedCalc package 20.104 software (MedCalc Software Ltd., Ostend, Belgium). The correlation coefficient between the level of PCSK9-Lp(a) complexes and the concentration of total cholesterol (r = 0.40) [41] was used to estimate the sample size needed to achieve adequate statistical power for the current study. Based on a correlation coefficient (r = 0.40), at an α of 0.05, a sample size of 61 patients was required to achieve a power of 90%. The data are presented as a median (25th, 75th percentile). The Mann–Whitney U test and Fisher’s exact two-tailed test were used in comparisons of independent groups. Spearman’s test was used for correlation analysis. A linear and multivariate linear regression were performed to identify the association between the studied parameters. The differences were considered statistically significant at *p* < 0.05.

## 3. Results

The clinical and immunological characteristics of the patients with coronary atherosclerosis and the control group are shown in Table 1.

**Figure 1:**
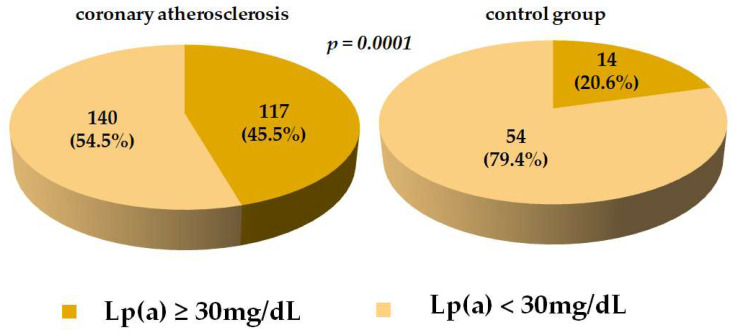
The proportion of patients with a level of Lp(a) ≥ 30 mg/dL in coronary atherosclerosis and controls groups. Data are presented as n (%). Abbreviations: Lp(a) = lipoprotein(a).

Fifty percent of the patients in the coronary atherosclerosis group had three- or multivessel coronary artery disease, 75% of the patients had a history of myocardial infarction, 82% of the patients underwent coronary stenting, and 21% of the patients underwent coronary artery bypass surgery. The proportion of patients with hyperLp(a) (≥30 mg/dL) in the atherosclerosis group was two times higher than in the controls (Figure 1).

**Figure 2:**
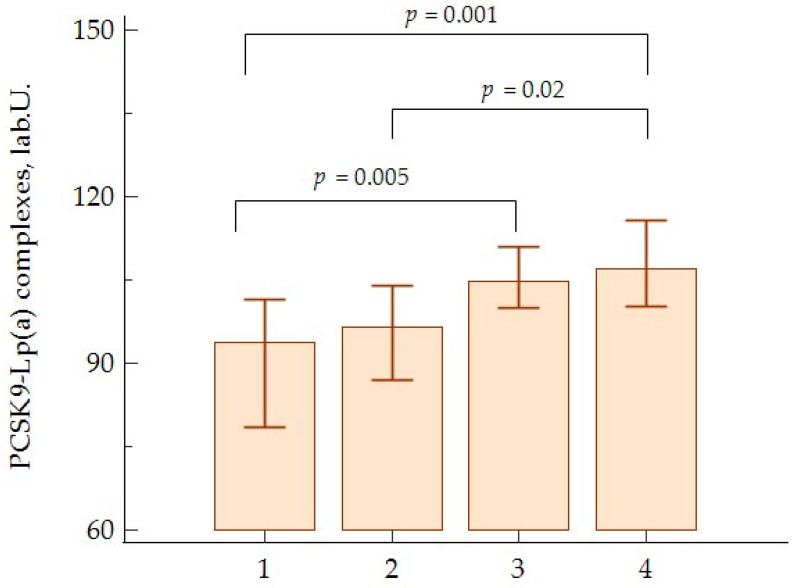
Levels of PCSK9-Lp(a) complexes according to monocyte quartiles in patients with atherosclerosis.

In the patients with atherosclerosis, the PCSK9-Lp(a) complexes levels were associated with higher quartiles of absolute monocyte values (Figure 2).

We did not observe correlations between the level of Lp(a) and the concentration of PCSK9-Lp(a) complexes, nor between the level of Lp(a) or PCSK9 and the total number of monocytes in both groups. A slight positive correlation between the concentration of PCSK9-Lp(a) complexes and the absolute level of monocytes was obtained (r = 0.20, *p* = 0.002) in the patients with atherosclerosis due to intermediate monocyte subsets (r = 0.33, *p* = 0.04). There were no correlations between the level of Lp(a), the concentration of PCSK9-Lp(a) complexes and the classical and non-classical monocyte subsets.

In the patients with atherosclerosis, a positive correlation was found between the PCSK9-Lp(a) complex levels and blood monocyte counts, predominantly in the hyperLp(a) group (>30 mg/dL) (r = 0.24, *p* = 0.01).

According to the regression analysis, both the PCSK9-Lp(a) complexes concentration and BMI were related to the absolute number of blood monocytes (Table 2) in the patients with atherosclerosis. When the age, hypertension, diabetes mellitus and Lp(a) and LDL-C levels were entered into a multivariate regression model, the above relationships remained independent and statistically significant (Table 2).

The positive correlation between the concentration of PCSK9 and the level of Lp(a) was found in patients with atherosclerosis (r = 0.31, *p* = 0.001) and in controls (r = 0.54, *p* = 0.016).

## 4. Discussion

Elevated Lp(a) concentrations are now recognized as a genetic risk factor for atherosclerotic cardiovascular disease and as a major residual lipid risk factor [43,44]. Inflammation is another major contributor to atherogenesis, with a confirmed role in the development of cardiovascular complications in patients achieving the target LDL cholesterol levels [45]. Despite the obvious role of both dyslipidemia and inflammation as key determinants of atherogenesis, the diversity and sequencing of inflammatory processes and the contribution of atherogenic lipoproteins to chronic inflammation in atherosclerosis is an extremely important area for investigation.

In this study, we analyzed the levels of Lp(a), PCSK9 and circulating PCSK9-Lp(a) complexes as well as the frequency of circulating monocytes in patients with stenotic coronary atherosclerosis. Age-matched patients without stenotic atherosclerosis of the coronary and peripheral arteries were included as a control group. In order to exclude the possible influence of gender differences on the indices analyzed, only male patients were included. There were no differences in the monocyte levels between the groups, but there was a tendency for the median monocyte level to increase in the group with coronary atherosclerosis. The lack of statistically significant differences can be explained by the inclusion of patients with stable coronary atherosclerosis without an active inflammatory process at the time of enrollment.

The proportion of patients with hyperLp(a) was two times higher in the group with stenotic coronary atherosclerosis than in the control group (45.5% vs. 20.6%), but no differences in monocyte levels were observed. No apparent correlation was found between the total monocyte content and Lp(a) concentration, which is consistent with previous studies [24]. A positive correlation between the blood Lp(a) and PCSK9 concentrations was observed in both the atherosclerotic and control patients, confirming our previous findings in patients with hypercholesterolemia [41]. However, no correlation was detected between the Lp(a) and circulating PCSK9-Lp(a) complexes.

Our observation that the levels of circulating PCSK9-Lp(a) complexes are associated with the blood monocyte levels was entirely novel. This finding was specific only to patients with stenotic atherosclerosis and not the patients without atherosclerotic lesions in any of the vascular basins.

The level of PCSK9-Lp(a) complexes was independently associated with the absolute number of peripheral blood monocytes in the patients with stenotic atherosclerosis, according to single-factorial and multivariate linear regression adjusted for other risk factors such as age, body mass index, Lp(a) and LDL-C concentrations and the presence of hypertension and diabetes.

The expansion of the monocyte population was due to an increase in the number of intermediate monocytes. According to published data, intermediate monocytes represent the most active pro-inflammatory cells [46,47]. An activating effect of the PCSK9-Lp(a) complexes on innate immunity appears to be realized in atherosclerosis. This may be due to both the phenotypic difference of apoprotein(a) and the ability of monocyte activation. The results of our previous study showed a redistribution of monocyte subpopulations towards an increased number of non-classical monocytes in hyperLp(a) patients. The combination of elevated Lp(a) and intermediate monocyte levels was associated with a significant increase in the odds of severe stenotic lesions in all three coronary arteries [24]. According to our observational study, monocyte levels above the median accompanied by Lp(a) concentrations above 30 mg/dL were associated with a 2.7-fold increased risk of cardiovascular events in patients with early-onset coronary artery disease [48].

There is evidence that hyperLp(a) is associated with monocyte activation. The monocytes isolated from the blood of patients with hyperLp(a) showed an increased capacity for transendothelial migration [49]. Transcriptome analysis of the CD14+ monocytes from patients with elevated Lp(a) levels before and after treatment with AKCEA-APO(a)-LRx antisense oligonucleotides, inhibiting apo(a) synthesis, showed a significant decrease in monocyte pro-inflammatory gene expression, leading to a reduction in monocyte transendothelial migration and decreased expression of Toll-like receptor 2 and the chemokine receptors CCR2 and CX3CR1 [50]. These observations suggest an additive contribution of Lp(a) and immune cells to the progression of atherosclerosis and the development of cardiovascular complications. However, a clear understanding of the mechanism of this phenomenon is lacking.

PCSK9 is a key regulator of apoB-containing lipoprotein metabolism through its ability to induce lysosomal degradation in internalized LDL receptors. According to a 15-year prospective cohort study, PCSK9 blood concentrations were associated with the development of cardiovascular complications even after adjustment for traditional risk factors, including LDL cholesterol levels [26].

There is evidence that PCSK9 has pro-inflammatory properties independent of LDL metabolism. In addition to the predominant expression of PCSK9 by hepatocytes, it is also expressed by cells of the vascular wall (including smooth muscle cells, endothelium and macrophages) [30,31,32]. PCSK9 suppresses the expression of ABCA1 transporter by macrophages, thereby inhibiting reverse cholesterol transport [51]. PCSK9 increases the macrophage expression of scavenger receptors, particularly CD36 and lectin-type oxidized LDL receptor 1-1 [52], which may contribute to foam cell differentiation and the growth of atherosclerotic lesions. Cultivation of macrophages derived from donor blood monocytes and THP-1 monocyte line cells in the presence of recombinant human PCSK9 was associated with the induction of pro-inflammatory cytokine interleukin-1beta, interleukin-6, tumor necrosis factor, CXCL2 and monocyte chemotactic protein-1 mRNA synthesis. Clinical data from the ATHEROREMO IVUS study [53] on the association of PCSK9 levels with the extent of a necrotic core in atherosclerotic plaque indirectly support the in vitro data.

The relationship between PCSK9 and the phenotype and functional activity of peripheral blood monocytes in patients with familial hypercholesterolemia and healthy individuals has been previously studied. Monocyte expression of the monocyte chemotactic protein-1 receptor CCR2 was threefold higher in these patients. The monocytes from the patients showed an increased ex vivo migration capacity. Anti-PCSK9 monoclonal antibody therapy was associated with suppression of inflammation by reducing CCR2-mediated monocyte migration [54]. Smooth muscle cell-secreted PCSK9 has been shown to reduce LDL receptor exposure on monocytes and increase LDL-mediated CCR2 expression by monocytes [55]. Krychtiuk K.A. [56] demonstrated a positive correlation between the percentage of classical monocytes and a negative correlation between the relative content of non-classical monocytes and PCSK9 blood levels in patients receiving statins. However, patients with PCSK9 levels above the median showed higher relative values of classical monocytes and lower values of non-classical monocytes.

Previously, Tavori et al. [38] demonstrated that the major pool of lipoprotein-associated PCSK9 resides in a complex with Lp(a). Accumulating data on the immunomodulatory effects of PCSK9 provided the rationale to investigate the relationship between Lp(a)-associated PCSK9 levels (PCSK9-Lp(a) complex) and the number and composition of circulating monocytes.

## 5. Study Limitation

Our study has some limitations. The retrospective study design did not allow assessing the causal relationship between the studied parameters and the development of atherosclerosis. Unlike the patients in the main group, the patients in the control group did not take statins. Due to the possible effect of statins on the studied parameters, statistical analysis and relationship assessment were performed separately within each group. We were unable to include a group of patients without atherosclerosis of coronary and peripheral arteries taking statins.

## 6. Conclusions

Here, we demonstrated that the level of the PCSK9-Lp(a) complexes (but not Lp(a)) directly correlates with the level of circulating monocytes. The described correlation was only observed in the group of patients with atherosclerosis and not in the control group. The level of PCSK9-Lp(a) complexes was independently associated with the absolute number of peripheral blood monocytes in patients with atherosclerosis, according to single and multivariate linear regression including other factors such as age, body mass index, hypertension, diabetes and the Lp(a) and LDL-C levels. The independent direct relationship between the level of circulating PCSK9-Lp(a) complexes and the absolute content of blood monocytes suggests additional molecular and cellular mechanisms of high atherogenicity of Lp(a) as a transporter of immunologically active molecules, particularly PCSK9. Further studies are required to determine the pathogenetic contribution of PCSK9-Lp(a) complexes to the development of atherosclerosis.

## Figures and Tables

**Table 1:** Clinical characteristics and immunological parameters in patients with coronary atherosclerosis and in the control group.

Parameter	Coronary Atherosclerosis Group (*n* = 257)	Control Group (*n* = 68)
Age (years)	60 (53; 66)	60 (53; 65)
Smoking	166 (64)	38 (56)
Arterial hypertension	213 (83)	50 (74)
Myocardial infraction	193 (75)	no
Coronary atherosclerosis severity		
1 vessel	52 (20)	no
2 vessels	77 (30)	no
3 and multivessel	128 (50)	no
CABG	53 (21)	no
Coronary stenting	210 (82)	no
Type 2 diabetes	61 (24)	11 (16)
Aspirin	230 (89)	55 (81)
ACEI or ARB	200 (78)	48 (70)
β blockers	228 (88)	55 (81)
Diuretics	64 (25)	14 (21)
Calcium channel blockers	79 (31)	15 (22)
Glucose (mmol/L)	5.5 (5.0; 6.1)	5.2 (4.6; 6.2)
BMI (kg/m^2^)	29.0 (26.0; 32.0)	28.5 (27.0; 32.0)
Obesity	106 (41)	32 (47)
TC (mmol/L)	3.8 (3.2; 4.5) *	4.8 (3.9; 5.9)
TG (mmol/L)	1.5 (1.1; 2.1)	1.5 (0.9; 2.0)
HDL-C (mmol/L)	1.1 (0.9; 1.3)	1.2 (1.0; 1.5)
LDL-C (mmol/L)	2.0 (1.5; 2.6) *	2.8 (2.0; 3.7)
LDL-C corr (mmol/L)	1.8 (1.3; 2.4) *	2.7 (1.8; 3.5)
Lp(a) (mg/dL)	23.0 (9.1; 73.3) *	10.7 (4.7; 25.0)
PCSK9 (ng/mL)	288.0 (237.4; 361.4) *	214.7 (161.2; 300.0)
PCSK9-Lp(a) (lab.U.)	101 (82; 118)	92 (81; 110)
Leukocytes (10^9^/L)	7.6 (6.3; 8.7)	7.1 (6.1; 8.4)
Monocytes (10^6^/mL)	0.59 (0.45; 0.72)	0.55 (0.44; 0.64)
Classical monocytes (10^3^/mL)	360.1 (293.2; 473.1)	385.2 (297.5; 480.2)
Intermediate monocytes (10^3^/mL)	41.3 (24.4; 67.0)	33.6 (23.7; 53.5)
Non-classical monocytes (10^3^/mL)	83.2 (68.1; 126.3)	92.6 (71.4; 134.7)

Data are presented as median (25%; 75%) or *n* (%). * *p* < 0.05 compared with the control group. Abbreviations: CABG = coronary artery bypass surgery, BMI = body mass index, TC = total cholesterol, TG = triglycerides, HDL-C = high-density lipoprotein cholesterol, LDL-C = low-density lipoprotein cholesterol, LDL-Ccorr = LDL-C corrected to the level of Lp(a) cholesterol, Lp(a) = lipoprotein(a) and PCSK9 = proprotein convertase subtilisin/kexin type 9.

**Table 2:** Linear regression analysis of the association between monocyte levels (10^6^/mL) with PCSK9-Lp(a) complexes, lipids and traditional atherosclerosis risk factors.

Parameter	Simple		Multiple	
	Coefficient	*p*-Level	Coefficient	*p*-Level
PCSK9-Lp(a) complexes	0.23	0.0004	0.27	<0.0001
BMI	0.19	0.003	0.25	0.0001
Age	−0.02	<0.0001	0.01	0.856
Hypertension	−0.014	-	−0.07	0.247
Diabetes	−0.047	-	0.02	0.761
Lp(a)	−0.008	-	−0.01	0.876
LDL-C	−0.073	-	0.11	0.087

Abbreviations: BMI = body mass index, LDL-C = low-density lipoprotein cholesterol and Lp(a) = lipoprotein(a).

## Data Availability

The data presented in this study are available upon request from the corresponding author.
